# Anatomical categorization of isolated non-focal dystonia: novel and existing patterns using a data-driven approach

**DOI:** 10.3389/dyst.2023.11305

**Published:** 2023-06-08

**Authors:** J. R. Younce, R. H. Cascella, B. D. Berman, H. A. Jinnah, S Bellows, J. Feuerstein, A. Wagle Shukla, A. Mahajan, F. C. F. Chang, K. R. Duque, S. Reich, S. Pirio Richardson, A. Deik, N. Stover, J. M. Luna, S. A. Norris

**Affiliations:** 1 Department of Neurology and Biomedical Research Imaging Center, University of North Carolina at Chapel Hill, Chapel Hill, NC, United States,; 2 School of Medicine, Washington University, St. Louis, MO, United States,; 3 Department of Neurology, Virginia Commonwealth University, Richmond, VA, United States,; 4 Department of Neurology, Emory University, Atlanta, GA, United States,; 5 Department of Human Genetics, Emory University, Atlanta, GA, United States,; 6 Department of Neurology, Baylor College of Medicine, Houston, TX, United States,; 7 Department of Neurology, University of Colorado Anschutz Medical Campus, Aurora, CO, United States,; 8 Department of Neurology, University of Florida, Gainesville, FL, United States,; 9 Rush Parkinson’s Disease and Movement Disorders Program, Rush University Medical Center, Chicago, IL, United States,; 10 Movement Disorders Unit, Neurology Department, Westmead Hospital & Sydney Medical School, University of Sydney, Sydney, NSW, Australia,; 11 James J. and Joan A. Gardner Family Center for Parkinson’s Disease and Movement Disorders, Department of Neurology, University of Cincinnati, Cincinnati, OH, United States,; 12 Department of Neurology, University of Maryland, Baltimore, MD, United States,; 13 Department of Neurology, University of New Mexico and New Mexico VA Healthcare System, Albuquerque, NM, United States,; 14 Parkinson Disease and Movement Disorders Center, Department of Neurology, University of Pennsylvania, Philadelphia, PA, United States,; 15 Department of Neurology, Heersink School of Medicine, The University of Alabama at Birmingham, Birmingham, AL, United States,; 16 Department of Radiology, School of Medicine, Washington University, St. Louis, MO, United States,; 17 Department of Neurology, School of Medicine, Washington University, St. Louis, MO, United States

**Keywords:** dystonia, clustering, segmental, non-focal, Dystonia Coalition

## Abstract

According to expert consensus, dystonia can be classified as focal, segmental, multifocal, and generalized, based on the affected body distribution. To provide an empirical and data-driven approach to categorizing these distributions, we used a data-driven clustering approach to compare frequency and co-occurrence rates of non-focal dystonia in pre-defined body regions using the Dystonia Coalition (DC) dataset. We analyzed 1,618 participants with isolated non-focal dystonia from the DC database. The analytic approach included construction of frequency tables, variable-wise analysis using hierarchical clustering and independent component analysis (ICA), and case-wise consensus hierarchical clustering to describe associations and clusters for dystonia affecting any combination of eighteen pre-defined body regions. Variable-wise hierarchical clustering demonstrated closest relationships between bilateral upper legs (distance = 0.40), upper and lower face (distance = 0.45), bilateral hands (distance = 0.53), and bilateral feet (distance = 0.53). ICA demonstrated clear grouping for the a) bilateral hands, b) neck, and c) upper and lower face. Case-wise consensus hierarchical clustering at k = 9 identified 3 major clusters. Major clusters consisted primarily of a) cervical dystonia with nearby regions, b) bilateral hand dystonia, and c) cranial dystonia. Our data-driven approach in a large dataset of isolated non-focal dystonia reinforces common segmental patterns in cranial and cervical regions. We observed unexpectedly strong associations between bilateral upper or lower limbs, which suggests that symmetric multifocal patterns may represent a previously underrecognized dystonia subtype.

## Introduction

Isolated dystonia may affect any distribution of the body in a focal or non-focal pattern (including segmental, multifocal, generalized, or hemidystonia), with subtypes categorized by the pattern of body region involvement. For clinicians and researchers alike, classification of the affected body distribution affords value in guiding therapies, monitoring spread over time, and informing care of associated non-motor features such as pain and psychiatric symptoms [1–4]. However, inconsistencies categorizing involved body regions may compromise the intended purpose [5–7]. For instance, recent studies variably classify dystonia of the shoulder plus a contiguous region as focal *versus* segmental, depending on which contiguous body regions are involved [8, 9]. This inconsistency prompted recommendations to alter the consensus guideline’s definition of focal cervical dystonia for application in future studies [6, 7]. Knowledge regarding which body regions are commonly involved in combination may assist with future similar classification guidelines with respect to dystonia involving more than one body region.

Focal cervical dystonia is thought to be the most common focal site in adult-onset dystonia [10–12], where generalization rarely occurs [13–16]. Dystonia in combinations of body regions such as upper or lower face, jaw, tongue, larynx, limbs or trunk are most frequently described in terms of anatomical contiguity (e.g., segmental), and reports of multifocal dystonia (i.e., dystonia in non-contiguous body regions) are limited [8, 13, 17]. Rare observance of multifocal dystonia may reflect *a priori* assumptions of dystonia distribution, commonly taught eponymous syndromes (e.g., Meige syndrome), and variability of scope and granularity in the studies that detect affected body regions. Analyzing the relationship of dystonia co-occurrence across body regions using purely data-driven analysis methods reduces some sources of variability and bias, and thus has the potential to reveal previously unrecognized patterns of non-focal dystonia.

Given the established value of defining body distributions affected by dystonia, we aimed to apply an empirical approach for guiding future categorization of non-focal subtypes. Specifically, we applied a data-driven approach to elucidate the frequency and co-occurrence of multiple pre-defined body regions exhibiting dystonia on examination in the Dystonia Coalition (DC) database, the largest standardized, multicenter cohort of patients with isolated dystonia ever assembled. We hypothesized that by identifying body regions with common dystonia co-occurrence, meaningful patterns would emerge. A better understanding of the relationship between body regions commonly affected in isolated non-focal dystonia will improve future consensus classification that may improve design and implementation of future studies, guide development of more sensitive measures, and improve the understanding of pathophysiologic mechanisms [18, 19].

## Methods

### Consent and protocol

Data were obtained from the DC, an ongoing multicenter international project aimed to delineate the clinical features and natural history of isolated dystonia. Methodological components of the DC are described elsewhere [20, 21]. For our purpose here, participants included in analyses were recruited by 57 movement disorders specialists at 43 sites across North America, Europe, and Australia. All investigators were trained to obtain and input standardized data (including examination of body regions affected by dystonia) to a centralized database. All participants gave written informed consent at the recruiting site according to the Declaration of Helsinki and The Common Rule. For the current study, the analysis of de-identified aggregate data was further approved by the Washington University Human Research Protection Office. All available DC data from 1/5/2011 to 10/1/2021 were filtered for inclusion (*n* = 3,240). For individuals with multiple evaluations over time, only data from the first DC visit were used for consistency.

### Participants

For inclusion in the DC, participants had to be at least 18 years old with a diagnosis of isolated dystonia [1]. Participants must not have acquired, combined or functional dystonia. Dystonia may affect any body region and be treated medically or surgically without exclusion. Genetic causes of isolated dystonia, when known, were not excluded.

For this study we only included participants with more than one of eighteen pre-defined body regions affected by dystonia (i.e., non-focal dystonia). These body regions included upper face, lower face, tongue, jaw, larynx, neck, left and right shoulders, left and right upper arms, left and right hands, trunk, pelvis, left and right upper legs, and left and right feet. As nearly all participants with upper or lower face dystonia were bilateral, right and left were combined for upper and lower face dystonia. Participants with only one affected body region at study enrollment were excluded. This resulted in the inclusion of 1,618 participants meeting these criteria. As the goal of this study was to assess co-association of dystonia affecting various body regions identified on examination, we did not include expert clinician classification of affected body distribution to define non-focal dystonia [5, 7].

### Clinical assessments

Expert movement disorder clinicians conducted a standardized neurological examination designed to elicit dystonia for all participants enrolled in the DC. The enrolling clinician documented the presence or absence of dystonia across the eighteen pre-defined body regions listed above. Other examination components performed for the DC were not included in this analysis.

In an effort to minimize confounding effects of handedness, we applied self-reported handedness data collected in the standardized assessment form by converting the laterality of any body region identified as “right/left” to “dominant/non-dominant.” For example, if someone self-identified as left-handed, then we relabeled the left shoulder, upper arm, hand, upper leg and feet as “dominant.” For purposes of this conversion, ambidextrous cases (*n* = 54, 3.3%) were treated as left-handed, similar to other studies. This was done for several reasons, including evidence of greater degree of ambidexterity among left-handed *versus* right-handed individuals as well as similarity of hemispheric language dominance patterns in ambidextrous individuals to left-handed individuals [22–24]. Handedness was carried through to all body regions to permit analysis of ipsilateral and contralateral effects on anatomical distributions of dystonia while controlling for dominant hand.

### Statistical analysis

#### Overview of general approach

The analytic approach included construction of frequency tables, variable-wise analysis using hierarchical clustering and independent component analysis (ICA), and case-wise consensus hierarchical clustering. This overall strategy was designed to assess the co-occurrence of dystonia across multiple body regions with data-driven analyses. Hierarchical clustering was employed due to a lack of *a priori* knowledge regarding the number of clusters in the dataset. We applied hierarchical clustering to variable-wise data to provide an overview of associations between body regions based on distance, then applied ICA to graphically represent the resulting body region groupings via extraction of independent signals. For additional granularity regarding associations between body regions, we clustered case-wise data using a consensus hierarchical clustering approach to estimate optimal cluster count and improve cluster stability.

Descriptive statistics and frequency analysis were performed in IBM SPSS Statistics 28 and hierarchical clustering and ICA were performed in R (version 4.1.2) [25].

### Clustering

We performed variable-wise hierarchical clustering of binary data representing the presence or absence of dystonia across eighteen pre-defined body regions by constructing an asymmetric binary distance matrix followed by agglomerative (bottom-up) hierarchal clustering using group average of the asymmetric binary distance. Subsequently, we performed consensus hierarchal clustering by case, a technique permitting quantitative assessment of cluster stability, number and membership [26]. Consensus hierarchical clustering by case was performed using the ConsensusClusterPlus package in R [27]. To assist with determination of optimal cluster number (k), consensus cumulative distribution function (CDF) was evaluated. We assessed CDF area under the curve plots using the elbow method to identify an optimal range of k values, where k = 9 was chosen via inspection of clustering plots for inclusion of all descriptively valuable clusters. Proportion of cases with dystonia in each body region was then generated by cluster. To ensure that clustering was not unduly affected by a single major data contribution site, we ran consensus clustering in a leave-one-site-out approach to exclude each of the eight sites each contributing to greater than 5% of total data. We visually examined the resulting CDF plots for the analyses performed with each site held out which confirmed no major deviation from clustering behavior for the complete data set (Supplementary Figures S1, S2).

### Independent component analysis

We used ICA to conduct an exploratory visualization of association of body regions by independent component. We performed ICA using the fastICA package in R [28]. As is standard for fastICA analyses, data centering and whitening were performed followed by principal component analysis (PCA). Three components were selected to preserve major features with sufficient dimensionality reduction for visualization. A 3-dimensional plot in component space was generated to visualize body region grouping.

## Results

### Participant characteristics and dystonia body region frequencies

Demographic characteristics for 1,618 participants who met inclusion criteria are reported in Table 1. Table 2 displays the frequency data of dystonia across all participants for each of the eighteen body regions analyzed. Neck (79.7% of cases) was the most common body region observed with dystonia, followed by upper and lower face (37.6% and 34.7%, respectively). Pelvis (2.3%) and upper leg (4.0%) were the body regions where dystonia was least frequently observed.

### Variable-wise hierarchical clustering

**FIGURE 1:**
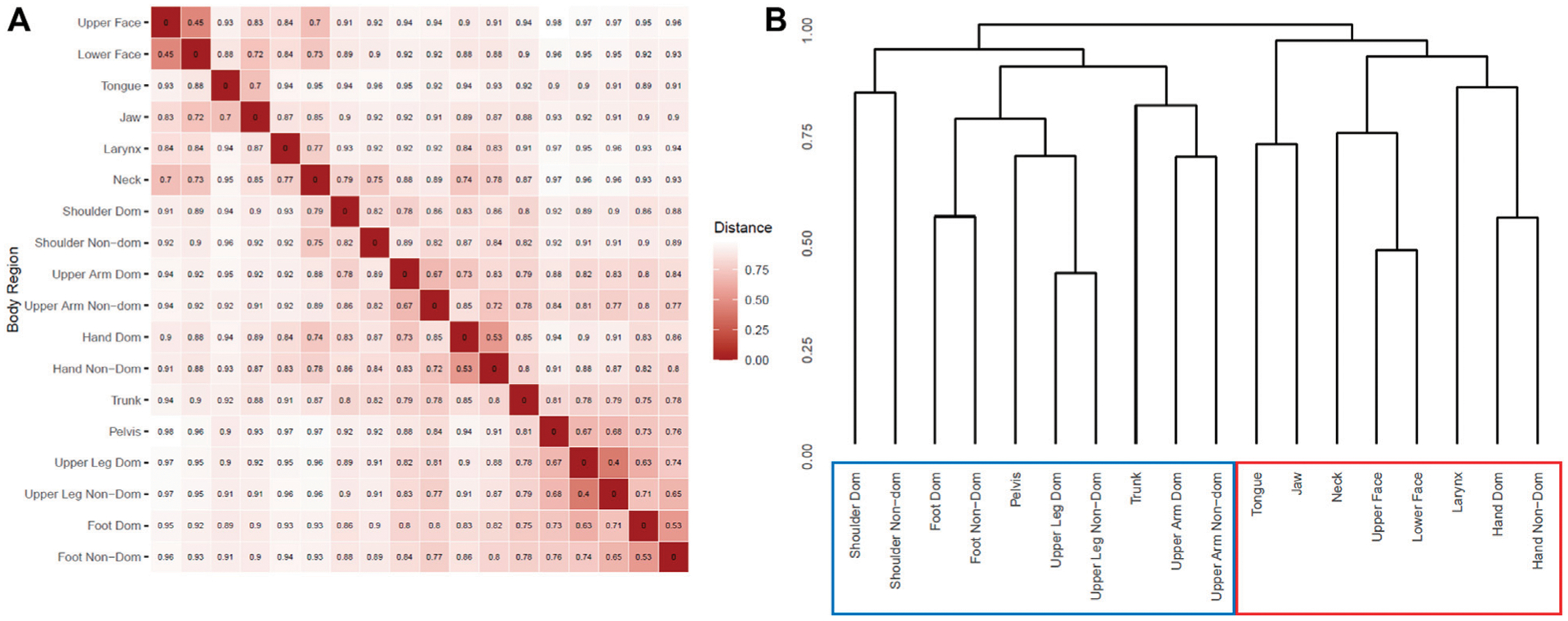
Hierarchical clustering by body region. **(A)** Asymmetric binary distance matrix for presence of dystonia in each reported body region. Smaller values represent shorter distances and thus more closely associated body regions. **(B)** Dendrogram result from agglomerative hierarchical clustering of body regions. Scale is in height which is generated from the distance matrix in **A**. “Dom”: dominant. “Non-Dom”: non-dominant. Red box highlights craniocervical + hand hierarchical group, while blue box highlights non-craniocervical hierarchical group.

The variable-wise binary distance matrix and derived dendrogram are shown in Figure 1. The strongest distance relationships were observed between bilateral upper legs (distance = 0.40), upper and lower face (distance = 0.45), bilateral hands (distance = 0.53), and bilateral feet (distance = 0.53). Most distances were large (over 0.7), even between regions expected to tightly associate, i.e., neck with shoulder, or hand with ipsilateral upper arm. The dendrogram (Figure 1B) generally split between craniocervical regions and the rest of the body, though both hand regions most closely associated with the craniocervical cluster, notably removed from the trunk cluster.

### Variable-wise ICA

**FIGURE 2:**
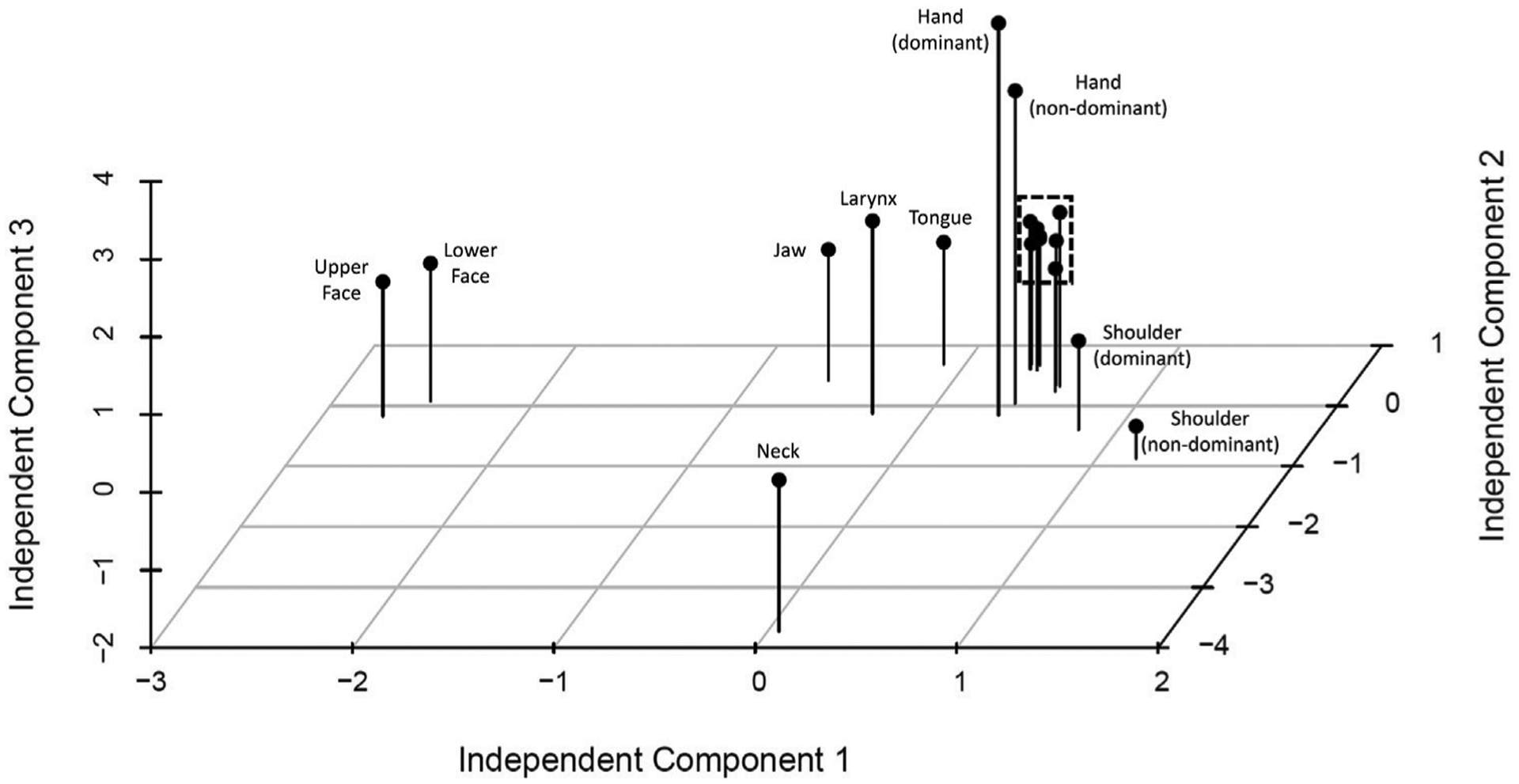
Independent component analysis by body region. 3 d scatterplot by independent components demonstrates clear grouping and separation from other body regions of a) neck, b) upper and lower face, and c) bilateral hands, with more subtle separation of d) jaw, larynx, and tongue, and e) bilateral shoulders. Tightly grouped regions indicated by dashed line are proximal regions including bilateral upper arms, bilateral upper legs, trunk, and pelvis.

The 3-dimensional scatterplot of body regions in component space is provided in Figure 2. Regions clearly separating themselves included 1) neck alone, 2) bilateral hands, and 3) upper face with lower face. Jaw with tongue and larynx also separated to a lesser degree, as did bilateral shoulders.

### Case-wise consensus hierarchical clustering

**FIGURE 3:**
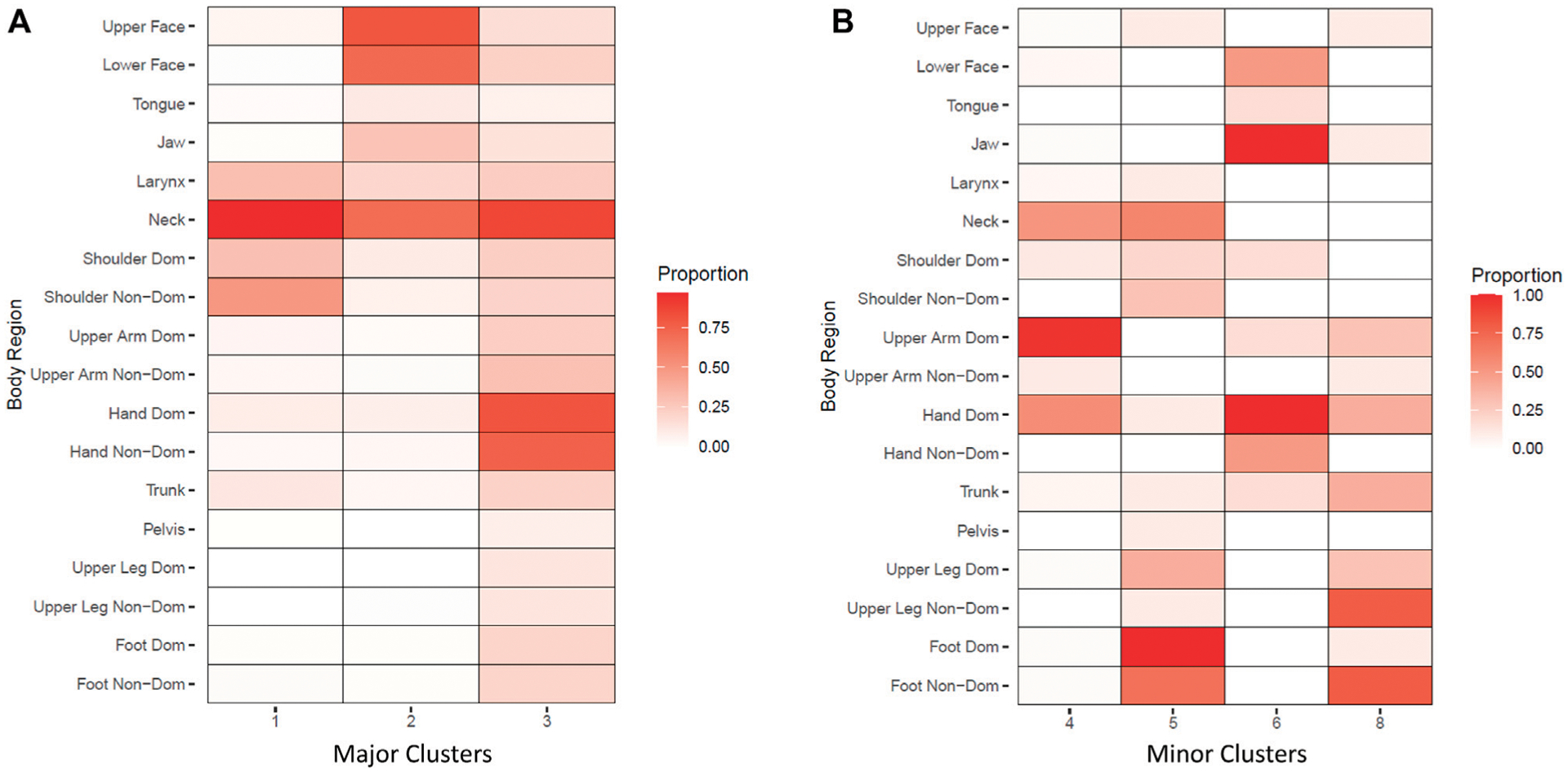
Consensus hierarchical clustering by case. Cell color indicates proportion of participants within each cluster where dystonia was observed in a particular body region. **(A)**: Major clusters (*n* > 100). **(B)**. *Minor* clusters (*n* < 100). Two clusters consisting of a single case were removed.

**FIGURE 4:**
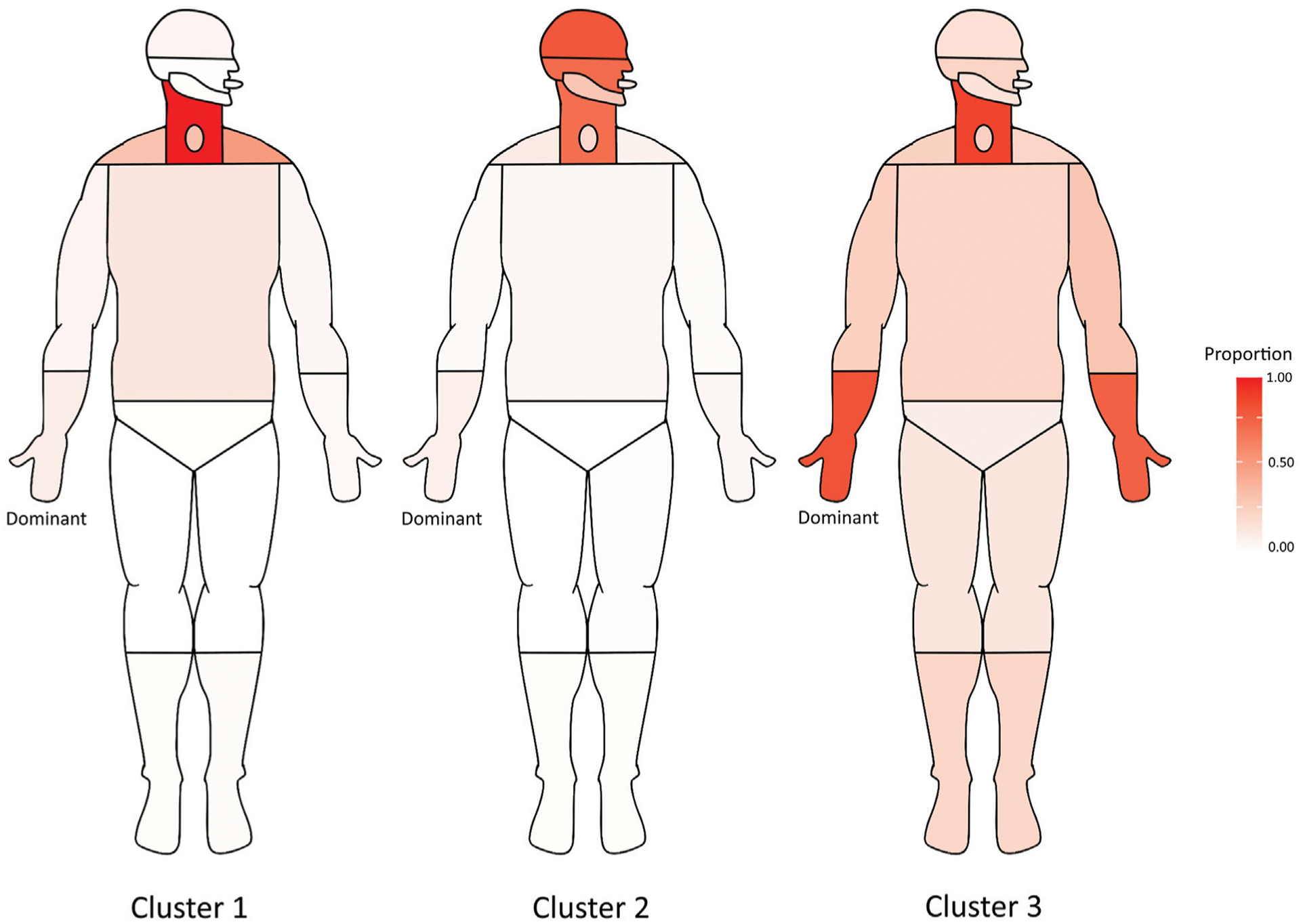
Anatomical representation of major consensus clusters (*n* > 100). Cell color indicates proportion of each cluster where dystonia was observed in a particular body region.

Visualization of clusters divided by size is shown in Figure 3, with major clusters plotted onto an anatomical representation in Figure 4. Of the three major clusters (*n* > 100), one primarily consisted of neck with some shoulder (cervical dystonia) and laryngeal involvement, another consisted primarily of cranial dystonia with less cervical involvement, and a third consisted primarily of hand dystonia of with cervical involvement. Two clusters included only a single participant and were thus removed.

## Discussion

Here we describe patterns derived from multiple data-driven methods applied to data extracted from the largest known multicenter study of isolated non-focal dystonia. Using variable-wise hierarchical clustering we observed a distinction between craniocervical dystonia and dystonia of other body regions, although interestingly hand dystonia clustered with craniocervical dystonia. With case-wise consensus hierarchical clustering, we observed three major clusters with four smaller minor clusters and two isolated distinct cases. Overall, we observe relatively tight associations between dystonia in upper and lower face, bilateral hands, and bilateral feet and upper leg. Some observed patterns are consistent with commonly reported patterns of dystonia, such as dystonia of the neck, shoulder, and larynx, and dystonia of the upper face with lower face and jaw involvement [13, 29]. These associations reinforce commonly reported segmental patterns such as cervical dystonia with laryngeal dystonia, and cranial dystonia including both upper and lower facial involvement [6, 7]. However, several patterns provided surprising associations. Specifically, we observed grouping of bilateral hands rather than hand with ipsilateral upper arm or shoulder. Observations in the feet and upper legs demonstrated similar preference for bilateral associations over ipsilateral foot and upper leg involvement. Independent component analysis reinforced this observation, with bilateral hands represented close together in component space but distant from the anatomically contiguous regions of upper arm and shoulder, and a similar pattern to a lesser degree in bilateral shoulders. Such grouping of non-contiguous regions contrasts with observations from prior prevalence studies that multifocal dystonias are less common than segmental, and much less common than focal dystonias [10–12]. These findings will be impactful to future to data collection and interpretation, possibly influencing future research approaches in non-focal dystonia.

With respect to categorizing dystonia body distribution, current consensus defines body regions involved as upper or lower cranial regions, cervical region, larynx, trunk, and upper limbs or lower limbs [1]. Consensus definitions regarding body distribution of dystonia include: focal (one region affected), segmental (two or more contiguous regions affected), multifocal (two or more non-contiguous regions affected with or without additional contiguous regions), hemidystonia (multiple body regions on one side affected), and generalized (trunk plus at least two other sites affected). The DC requires identification of specific body parts affected not specifically sub-categorized in the current consensus guidelines, including the jaw, tongue, shoulder, pelvis, upper arm, hand, upper leg and foot. Thus, there may be additional consideration needed to determine how to isolate or combine body regions when categorizing focal and non-focal dystonia for research purposes [8, 9, 13, 17]. To avoid loss of data granularity in our study, we did not group Dystonia Coalition-defined sub-regions together (e.g., hand with upper arm), thus allowing these sub regions to cluster together naturally. This implies the definition of “non-focal” within this study is not identical to current consensus criteria, i.e., hand here would be considered a separate region from upper arm [1]. The empirical tendency for bilateral hands to cluster together, rather than hand clustering with ipsilateral upper arm, suggests that these regions behave distinctly in terms of co-association with other body regions, and thus it may not be appropriate for dystonia involving both hand and upper arm to be considered “focal.” A similar tendency for bilateral feet to cluster together, rather than with ipsilateral upper leg, reinforces this observation. It is quite possible that increased anatomical data granularity in future studies, i.e., distinguishing between muscle groups that control individual joints, might further distinguish regions currently identified as “focal” vs. “segmental.” Specifically, EMG may provide improved quantitative analysis of muscles engaged above the region of phenotypic observation in assessing the extent of proximal to distal limb involvement.

The major hierarchical division between dystonia in craniocervical regions and dystonia in other body regions may have implications for underlying mechanisms, particularly in consideration of pathophysiologic or genetic etiologies. Craniocervical dystonias have been noted to share pathophysiological mechanisms, particularly at the level of trigeminal sensory disruption [30]. As more genetic etiologies are identified for dystonia, it has become clear that anatomic distribution relates strongly to genotype, with some genes (e.g., GNAL) typically causing a craniocervical-predominant syndrome, while craniocervical involvement is less frequent in others (e.g., TOR1A) [31]. It is possible that data-driven anatomic clusters may be useful for categorizing future genomic studies in dystonia by avoiding excessive “lumping” of dystonia classification and enhancing specificity when searching for potential pathogenic mutations or pathophysiologic mechanisms.

The surprising finding that hand dystonia associates most strongly with contralateral hand dystonia rather than adjacent upper arm or shoulder, and that the same pattern is found in the feet, may have conceptual implications for the dystonia mechanism. In non-focal dystonia, the concept of body region “spread” may be pathophysiologically considered as extension from one focus to a somatotopically proximate region within cortical or subcortical brain regions [13, 32, 33]. With recognition of symmetric bilateral involvement of distal extremities without observance of anatomical contiguity, neuroanatomical proximity cannot reasonably be invoked. One alternative possibility is that mirroring through interhemispheric crossing fibers, in cortex or elsewhere, is responsible for this phenomenon, which has previously been observed even in focal hand dystonia [34]. The notion of contiguity in dystonia may also need to be revisited, given observed differences in somatotopic organization between motor cortex and internal globus pallidus [35, 36]. A more distributed network may also be involved, particularly given evidence for involvement outside of basal ganglia and cortical structures, including cerebellar contributions to dystonic networks [37]. Furthermore, while those with bilateral hand dystonia had an increased rate of task-specific dystonia (36%, vs. 24% in the overall cohort) and tremor-predominance (28%, vs. 16% of the overall cohort), most with bilateral hand dystonia did not exhibit these features—noteworthy as tremor and task-specificity may have pathophysiological implications [38, 39].

In the cluster exhibiting most bilateral hand dystonia, Cluster 3, a wide variety of other body regions also exhibited dystonia to a lesser degree. While most members of this cluster would not meet strict consensus criteria for a generalized dystonia (i.e., there is no trunk involvement), there may be a predisposition of those in this cluster to develop dystonia in non-contiguous body regions (i.e., multifocal dystonia). This may be rooted in the “permissive condition” hypothesized to be necessary for the development of dystonia as a response to various insults [40, 41]. In this cohort we did not observe a distinct cluster representing generalized dystonia, with generalized cases clustering primarily with multifocal cases within Cluster 3. Among cases in Cluster 3, 21% were observed to have truncal involvement, with all truncal cases meeting criteria for generalized dystonia. It should also be noted that this rate of generalized dystonia may also represent a degree of under-recognition of truncal involvement, including cases presenting at an early stage of generalized dystonia. Although we did not remove cases with known genetic syndromes (i.e., TOR1A), our cohort was limited to those over 18, while a cohort including pediatric participants would likely include more genetically confirmed generalized dystonia. While current dystonia classifications emphasize contiguity of body regions, our approach suggests that non-contiguous syndromes (e.g., bilateral hand, multifocal dystonia) may be more prevalent than previously believed.

Although identification of dystonic body regions within the DC occurs according to strict criteria and in a standardized format, some potential for observer bias exists, as does inter-observer variability [21]. For example, observer bias for separating out shoulder dystonia when neck dystonia is observed (i.e., cervical dystonia), or not including upper arm dystonia where hand dystonia is observed, may affect these analyses and conclusions. Dystonia in upper arm, upper leg, pelvis and trunk are likely to be particularly susceptible to bias, as subtle dystonia in these regions may require more careful examination and not be as evident to all examiners as regions such as hands and face. Similarly, it is plausible that when dystonia is noted in one hand, there may be more focus on the contralateral hand and thus more probability of noting subtle dystonia there. To minimize these effects, we not only used a consensus clustering approach designed to reduce the effect of an aberrant data source on the overall result, but also used a leave-one-out approach with each major DC site to ensure that clustering structure was not unduly affected by a single rater or site. While this method limits the potential impact of a discordant rater on overall clustering, systematic perceptual issues are still a potential limitation of this methodology, as raters may be systematically more prone to note dystonia in areas like face and hands than in areas like upper arm, trunk, and pelvis. These limitations in granularity may also be impacted by the relative predominance of cranial and cervical dystonia in the DC cohort, even in this subgrouping of non-focal cases.

Data-driven classification of non-focal dystonia reinforces some commonly described patterns, including segmental cervical and cranial dystonic syndromes. However, converging evidence using multiple data-driven analyses suggest that symmetric multifocal dystonia may be more common than previously recognized. The tendency for hands to group together rather than with their ipsilateral upper arm challenges the current consensus categorization of focality in dystonia, bringing to light considerations regarding recognition, classification, and pathophysiologic mechanisms of non-focal dystonia.

## Supplementary Material

Supplementary Material

## Figures and Tables

**TABLE 1:** Demographic and clinical characteristics of study population as reported to Dystonia Coalition.

Category		Participants (% total)
Gender	Female	1,146 (70.8%)
	Male	472 (29.2%)
Race	White	1,450 (89.6%)
	Non-white	153 (9.5%)
	Not reported/Unknown	15 (0.9%)
Handedness	Right	1,404 (86.8%)
	Left	155 (9.6%)
	Ambidextrous	54 (3.3%)
	Unknown	5 (0.3%)
Age at visit (years)		60.2 ± 13.0
Age at onset (years)		45.1 ± 16.8
Disease duration (years)		15.0 ± 13.0
BFM Total		11.1 ± 9.5
GDRS Total		12.6 ± 10.3

BFM: Burke-Fahn-Marsden Dystonia Rating Scale. GDRS, Global Dystonia Rating Scale. Relevant values reported in mean ± standard deviation.

**TABLE 2:** Percentage of participants in non-focal dystonia cohort with dystonia reported in each body region.

Body region	Percent reported (%)
Neck	79.7
Upper Face	37.6
Lower Face	34.7
Hand (dominant)	30.7
Hand (non-dominant)	22.6
Larynx	22.6
Shoulder (non-dominant)	21.6
Shoulder (dominant)	18.8
Jaw	15.6
Upper Arm (dominant)	13.2
Trunk	10.9
Upper Arm (non-dominant)	10.1
Foot (non-dominant)	7.1
Foot (dominant)	6.7
Tongue	6.5
Upper Leg (non-dominant)	4.0
Upper Leg (dominant)	3.9
Pelvis	2.3

As all participants had dystonia in multiple body regions, percentages do not sum to 100%. “Dominant” and “non-dominant” refer to the lateral relationship with self-reported dominant hand, as only handedness data was collected.

## Data Availability

The data analyzed in this study is subject to the following licenses/restrictions: Data is managed by the Dystonia Coalition. Requests to access these datasets should be directed to dystoniacoalition@emory.edu.
