# Supplementary material for: Anatomical categorization of isolated non-focal dystonia: novel and existing patterns using a data-driven approach
Source: Dystonia. Author manuscript; Available in PMC 2023 Nov 2. (PMC10621194; doi:10.3389/dyst.2023.11305)
Supplement: Supplementary Material [file NIHMS1907556-supplement-Supplementary_Material.docx]

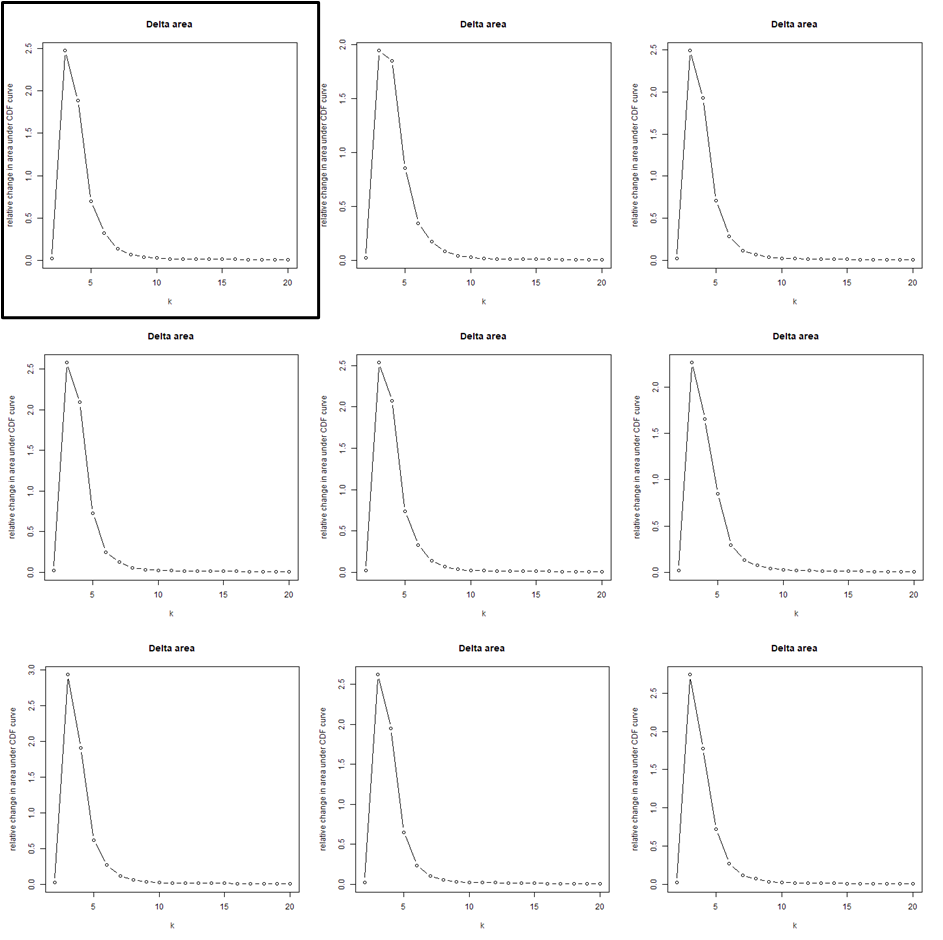


Supplementary Figure 1: CDF plots for all data (top left, black outline) and with data from each large site (n = 8, minimum 5% data contributed) held out. Clustering behavior remained grossly similar with each major site held out, with “elbow” identified between k = 6 and k = 9.


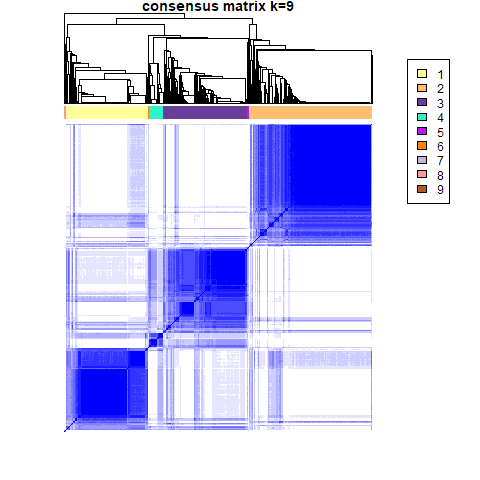


Supplementary Figure 2: Consensus hierarchical clustering by case at k = 9. Dendrogram and consensus matrix are displayed with cluster number labelled in color.
